# Community-Based Interventions to Improve HPV Vaccination Coverage among 13- to 15-Year-Old Females: Measures Implemented by Local Governments in Japan

**DOI:** 10.1371/journal.pone.0084126

**Published:** 2013-12-16

**Authors:** Hiroyuki Fujiwara, Yuji Takei, Yoshiki Ishikawa, Yasushi Saga, Shizuo Machida, Akiyo Taneichi, Mitsuaki Suzuki

**Affiliations:** 1 Department of Obstetrics and Gynecology, Jichi Medical University, Yakushiji, Shimotsuke, Tochigi, Japan; 2 Department of Health and Social Behavior, School of Public Health, The University of Tokyo, Hongo, Bunkyo-ku, Tokyo, Japan; Georgetown University, United States of America

## Abstract

The purpose of this study was to examine the effect of various community-based interventions in support of HPV vaccination implemented by cities and towns within Tochigi prefecture, Japan with a view to identifying useful indicators which might guide future interventions to improve HPV vaccination coverage in the prefecture. A postal questionnaire survey of all 27 local governments in Tochigi Prefecture was conducted in December 2010. All 27 responded, and 22 provided the exact numbers of the targeted and vaccinated populations of 13- to 15-year-old girls from April to December 2010. The local governments also answered questions on the type of interventions implemented including public subsidies, school-based programs, direct mail, free tickets and recalls. Local governments that conducted a school-based vaccination program reported 96.8% coverage for the 1^st^ dose, 96.2% for the 2^nd^ dose, and 91.2% for the 3^rd^ dose. Those that provided subsidies without school-based programs reported a wide range of vaccination rates: 45.7%–95.0% for the 1^st^ dose, 41.1%–93.7% for the 2^nd^ dose and 3.1%–90.1% for the 3^rd^ dose. Among this group, the combination of a free ticket, direct mail and recall was most effective, with 95.0% coverage for the 1^st^ dose, 93.7% for the 2^nd^ dose, and 90.1% for the 3^rd^ dose. The governments that did not offer a subsidy had the lowest vaccination coverage, with 0.8%–1.4% for the 1^st^ dose, 0.0%–0.8% for the 2^nd^ dose, and 0.1%–0.1% for the 3^rd^ dose. The results of this survey indicate that school-based vaccinations and public subsidies are the most effective method to improve HPV vaccination coverage; however, the combination of a free ticket, direct mail, and recalls with public subsidies are also important measures in increasing the vaccination rate. These data may afford important indicators for the successful implementation of future HPV vaccination programs.

## Introduction

Human papillomavirus (HPV) vaccination is an effective strategy for preventing uterine cervical cancer. The vaccination was first introduced in the United States in 2006 and spread across the world [1]. In 2013, two types of HPV vaccines are available in many countries: one is Gardasil^®^, a quadrivalent vaccine targeting HPV 6/11/16/18; and the other is Cervarix^®^, a bivalent vaccine targeting HPV 16/18 [1-4]. Targeting young adolescent females is recommended because mathematical models predict that vaccination significantly reduces HPV infection and associated disease among that population [5-7].

However, despite the need for HPV vaccination, few studies have focused on interventions to increase HPV vaccination coverage, except for school-based vaccination programs [8,9]. Moreover, it cannot be appropriate to refer to the evidence about interventions for other vaccines, such as measles, diphtheria-tetanus toxoid, poliomyelitis, hepatitis B and varicella, because these vaccines target children, not adolescents [10].

Since 2010, Japanese local governments at the level of cities and towns have been in charge of offering HPV vaccinations and deciding whether to provide public assistance and other interventions including school-based offerings, direct mail, recalls, and free tickets. As a result, each local government has a different combination of interventions: some local governments provide comprehensive interventions, while others require their residents to pay $500 for the completion of 3 doses.

Therefore, the purpose of this study was to examine the effect of community-based interventions for HPV vaccination that varies across local governments and to present data that might be helpful for planning future interventions to improve HPV vaccination coverage in the cities and towns of Tochigi prefecture, Japan.

## Methods

**Figure 1:**
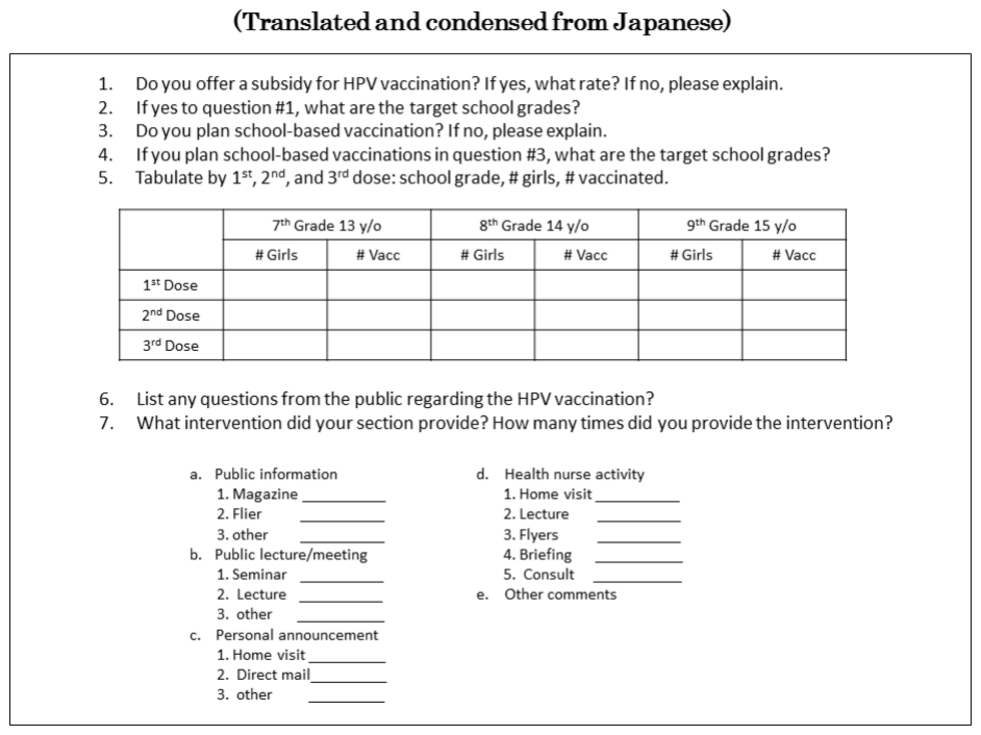
HPV Vaccination Survey. The HPV Vaccination Survey was sent to all 27 local government entities (cities and towns) in Tochigi prefecture, Japan in October 2010 requesting data for April through December 2010. Complete responses were received from 22 local governments. Supplementary data was obtained by follow-up telephone surveys.

A postal questionnaire survey of 27 local governments in Tochigi Prefecture was conducted in December 2010 (Figure 1). The Health Promotion Section of all 27 local governments responded, and 22 provided the exact numbers of the targeted and vaccinated populations from April to December 2010 among 13- to 15-year-old females who were junior high school students. In Japan, the recommended age for receiving the HPV vaccination series is from 13 to 16 years old. When students in Japan enter high school, usually at age 16, a significant number opt to attend a school outside their home prefecture, and would be absent from the target population. If these students are vaccinated, they would be included in the vaccinated population because vaccinations are reported to their home jurisdiction. Junior high school students have no such option. Therefore, we chose to exclude high school students from our target to avoid a confounding effect on the results.

A second questionnaire was distributed in August 2012 only to determine the final number of the vaccinated population within Tochigi prefecture. The vaccinated populations from each survey were composites of all vaccinations from both government entities and private providers.

The targeted populations were determined by then-current enrollment records from junior high schools in the local government jurisdictions ordered by school grade. The vaccinated populations were compiled by the Health Promotion Sections of local governments from public and private reports of vaccinations which are required by law, and sorted by age.

The local governments also answered questions on the types of interventions implemented including public subsidies, school-based programs, direct mail, free tickets and recalls. *Public subsidies* come from local governments to allow the targeted population to be vaccinated free of charge. A s*chool-based program* refers to a mass vaccination that targets a student population. *Direct mail* aims to provide information about the HPV vaccine, procedures, the targeted population, subsidy amount, and available medical institutions. *Free tickets* focus on financial incentives that would benefit residents. *Recalls* are conducted through postal mail to inform the unvaccinated population of opportunities for receiving the vaccination.

**Figure 2:**
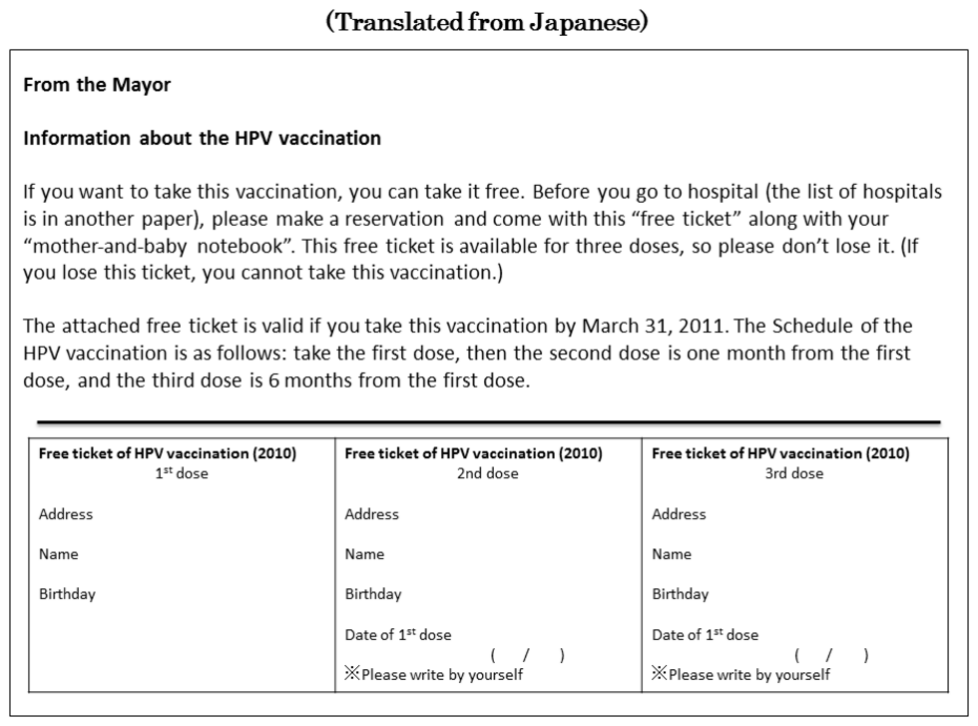
Direct Mail & Free Ticket. Direct mail, usually including 3 free tickets, were independently or cooperatively designed and mailed by the 27 local government entities in Tochigi prefecture, Japan. Direct mail was conducted once prior to the start of a vaccination program. The sample provided here is an anonymized translation from Japanese of a direct mail campaign with free tickets by one city that collaborated with two towns.

**Figure 3:**
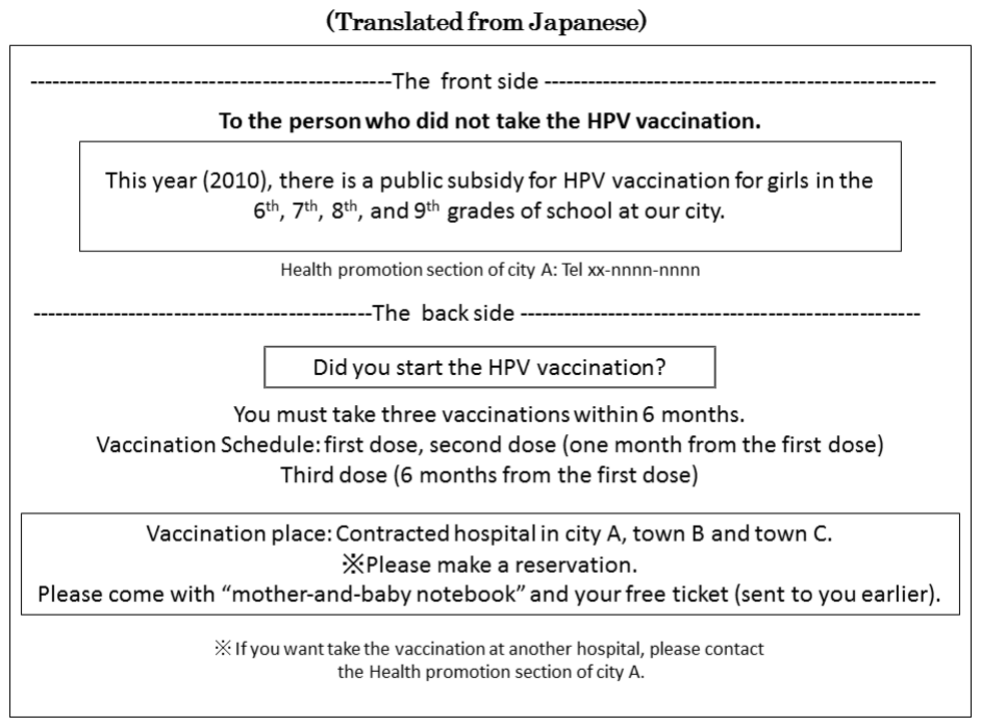
Recall. Recall notices were mailed once to people who after several months did not respond to the direct mail campaign by scheduling a vaccination. The sample provided here is an anonymized translation from Japanese of a recall notice by one city that collaborated with two towns.

The free ticket and direct mail (Figure 2) was sent just once prior to the series of vaccinations. If there was no response from the targeted person within several months, a single recall (Figure 3) was mailed. Direct mail and Recall notices contain just basic information such as immunization stage, available dates and places of immunization.

For the analysis, the 22 local governments that had provided the exact numbers of their targeted and vaccinated populations were divided into groups according to the combination of implemented interventions. This allowed comparison of the HPV vaccination coverage for the 1^st^, 2^nd^ and 3^rd^ doses. Statistical analyses were performed using the χ^2^ test.

Our study does not fall into the definition of research involving ”human subjects” according to the Japanese ethic guidelines, and the analysis herein involved the use of an anonymized publicly available dataset.

## Results

Of the 22 local governments in Tochigi Prefecture that provided the exact numbers of their targeted and vaccinated populations, 2 conducted a school-based vaccination program with subsidy, 12 provided a community-based vaccination program with subsidy, and 8 did not offer any type of subsidy. 

**Figure 4:**
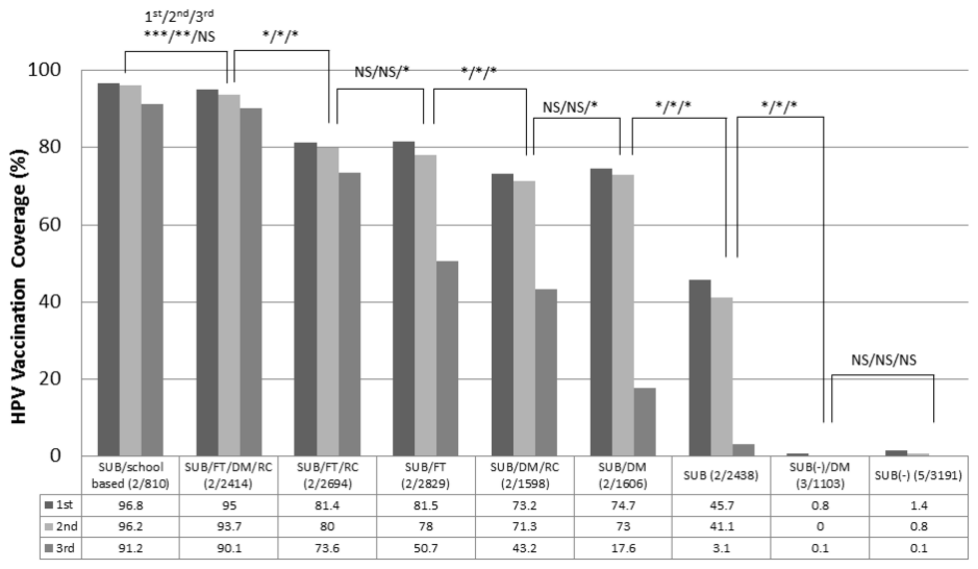
HPV vaccination coverage among 13- to 15-year-old females according to combinations of interventions. The local governments that conducted a school-based vaccination program with subsidy achieved the highest coverage: 96.8% for the 1^st^ dose, 96.2% for the 2^nd^ dose, and 91.2% for the 3^rd^ dose. SUB: subsidy, FT: free ticket, DM: direct mail, RC: recall, ( / ): Number of local government/total number of targeted population. *p<0.001, * *p<0.01, ***p<0.05, NS: not significant.

Figure 4 shows the coverage rates and the statistical comparison results of HPV vaccination according to combination of interventions. The local governments that conducted school-based vaccination programs targeted 810 students with a 100% subsidy. This represented all females then enrolled in junior high schools within their jurisdictions. These programs achieved the highest coverage rate: 96.8% for the 1^st^ dose, 96.2% for the 2^nd^ dose, and 91.2% for the 3^rd^ dose. Subsidy-based programs demonstrated a wide range of vaccination rates: 45.7%–95.0% for the 1^st^ dose, 41.1%–93.7% for the 2^nd^ dose and 3.1%–90.1% for the 3^rd^ dose. Among this group, the combination of a free ticket, direct mail and recall was most effective, with 95.0% coverage for the 1^st^ dose, 93.7% for the 2^nd^ dose, and 90.1% for the 3^rd^ dose. The local governments that did not offer any subsidy had the lowest vaccination coverage, with 0.8%–1.4% for the 1^st^ dose, 0.0%–0.8% for the 2^nd^ dose, and 0.1%–0.1% for the 3^rd^ dose. Table 1 shows the completion rates of the HPV vaccination by age. All of the ranges within the groups are smaller than 10%.

**Table 1:** Completion rates of HPV vaccination by targeted age according to combinations of interventions.

Combinations of Interventions (Number of Local Governments)	13 years old			14 years old			15 years old	
	Number of Targeted Population	Completion Rate (%)		Number of Targeted Population	Completion Rate (%)		Number of Targeted Population	Completion Rate (%)
School-based Vaccination Program with Subsidy (2)	NA	NA		78	97.4		732	90.6
Community-based Vaccination Program with Subsidy								
Free Ticket, Direct Mail and Recall (2)	783	89.8		822	90.4		809	90.0
Free Ticket and Recall (2)	901	73.5		893	73.8		900	73.4
Free Ticket (2)	925	45.7		961	52.4		943	53.7
Direct Mail and Recall (2)	524	35.9		509	47.7		565	45.8
Direct Mail (2)	538	14.7		535	16.1		533	22.0
No Intervention (2)	778	3.3		845	3.1		815	2.9
Community-based Vaccination Program without Subsidy								
Direct Mail (3)	374	0.0		379	0.3		350	0.0
No Intervention (5)	1041	0.3		1071	0.1		1079	0.0

## Discussion

To the best of our knowledge, this is the first study to examine the effects of different kinds of community-based interventions that vary across local governments in the same prefecture. This study was conducted solely within Tochigi prefecture, Japan. A prefecture is a sub-national political entity equivalent to a state in the United States and a province in other countries. Prefectures are further subdivided into cities and towns. According to the Tochigi Prefecture website, as of 2013 Tochigi prefecture has a population of 1,981,584 individuals with a total number of households numbering 773,739, an average family size of 2.56, and an average yearly income of $45,700.

The most important finding of the present study is that a community-based vaccination program can be as effective as a school-based vaccination program if the local government provides enough interventions with the combination of free tickets, direct mail, and recalls. The findings of our study also suggest that the vaccination coverage within a group of local governments offering subsidy significantly varies according to the combination of interventions used. The effect of the subsidy on the completion rate even vanished if the local government did not offer any other interventions. The substantial drop in the completion rate for the 3^rd^ dose of the vaccination series is surprising. It may be due to a combination of factors including: enduring pain and numbness from the first two doses dissuading a further dose, late staging of vaccination programs pushing the 5-month interval between the 2^nd^ and 3^rd^ doses outside of the survey period, and reporting variances among the 22 different local governments.

Consistent with previous studies, publicly funded school-based vaccination programs are an especially effective means for increasing the vaccination rate [11–13]. Particularly in Japan, local governments have made tremendous efforts to provide school-based vaccination programs. Since 1994, when the Preventive Vaccination Act was revised and all vaccinations became private as a rule, each local government had to create a new system by holding consultations in advance with the local medical association, the elementary school principals’ association, school nurses, parents, and others, to obtain approval from all quarters [13].

Therefore, it can be said that this study provides the first evidence that community-based interventions with the combination of free tickets, direct mail, and recalls can be a substitute for the school-based vaccination program. Since HPV vaccination requires three vaccination doses with intervals in between, it is expected that some people will not complete the series.

We found that coupons have had a stronger effect on the vaccination rate than direct mail. Distributing free tickets directly to individuals is a reliable means of informing people that private vaccinations are available free of charge. Moreover, the tickets themselves are in essence free money, which encourages people to use them and not waste them.

It is obvious that the most important method for increasing the vaccination rate is subsidizing the cost [14,15]. Public subsidies for HPV vaccination are common in many countries, and unique programs have been formed in effort to increase vaccination delivery [16–20]. In the present survey as well, local governments providing public subsidies were found to achieve higher vaccination rates than those without subsidies.

Although the first HPV vaccine was approved in the United States and in many other countries in 2006, Japan only approved its use in October 2009. Subsidies of some local governments began in April 2010 which is the start of the school year in Japan. Inasmuch as our survey commenced in April 2010 and the vaccination series requires 6 months to complete, we can be reasonably confident that there was no meaningful baseline data in the surveyed population.

In June 2013, The Japanese Ministry of Health, Labor, and Welfare decided to temporarily withdraw its recommendation regarding the use of HPV vaccines due to side effects such as long-term pain and numbness. The relationship between the vaccines and the symptoms remains unclear and public subsidies continued. However, if support of local governments is lost, even if vaccination is provided free of charge the coverage rate can be expected to fall dramatically, as our study demonstrates.

### Practical considerations

It is clear from the statistical analysis depicted in Figure 4 that the intervention with the best completion rates in our study is school-based with subsidy. The second-best intervention is free‑ticket plus direct‑mail and recall with subsidy. Compared to the former, the latter had lower completion rates in dose 1, dose 2, and dose 3 with differentials of 1.8%, 2.5%, and 1.1% respectively. Notwithstanding the statistical significance of this fact, it is noteworthy that of the twenty-two local government respondents, only two elected to utilize a school‑based intervention; and the population thus represented was only 810 students — the lowest population by far of any other intervention group. The population of the second‑best intervention group, which also comprised two respondents, was 2,414 students — three times as many as the school‑based group. Although school-based interventions showed a higher ratio of dose completions due to their captive populations, the second-best intervention strategy actually achieved more vaccinations due to its larger target population. School-based interventions in Japan are less likely to be employed by local governments due to massive complexities of political, logistical and legal issues, compounded by long lead times for startup. Furthermore, our study suggests that school‑based interventions tend to be utilized only in small towns. Thus, from a practical standpoint, the intervention strategy utilizing a subsidy, free-ticket, direct-mail, and recall, while statistically inferior, avoids the pitfalls of school-based interventions, and may well be the preferred choice for all but minimally populated cities and towns in future vaccination campaigns.

### Limitations

This study has a limitation in that the existence and effects of confounders on the vaccination rate were not known. Possible confounders could be age, vaccine history, area of residence, parental education, and family income [9]. As such, the causal effect of each intervention presented in this study could be biased. Future research should explore to what extent such unmeasured confounders play a role in analyzing the effects of interventions.

## Conclusion

In 2010 each Japanese local government had its own mixed intervention strategies for HPV vaccination, even within the same prefecture. Therefore, it was possible to compare the effects of various types of community-based interventions. Our study shows that interventions of school-based vaccination with public subsidy statistically achieve the highest vaccination coverage rate. However, from a practical standpoint, the combination of a free ticket, direct mail and recall with subsidy is an attractive option, only slightly less effective, which may be preferred by local governments with large populations. 

These data may yield important findings for improving current and implementing future HPV vaccination strategies in the community.
